# Genome-wide association study dissection of candidate genes for fleece traits in Inner Mongolia cashmere goats based on whole-genome resequencing data

**DOI:** 10.5713/ab.250631

**Published:** 2025-12-18

**Authors:** Huanfeng Yao, Silang Zhu, Rigan Xu, E′erke Ale′de, Yongbin Liu, Jinquan Li, Qi Lv, Ruijun Wang, Yanjun Zhang, Rui Su, Zhiying Wang

**Affiliations:** 1 College of Animal Science, Inner Mongolia Agricultural University, Hohhot, China; 2 Inner Mongolia Yiwei White Cashmere Goat Co., Ltd., Ordos, China; 3 Inner Mongolia Key Laboratory of Sheep & Goat Genetics Breeding and Reproduction, Hohhot, China; 4 Key Laboratory of Mutton Sheep & Goat Genetics and Breeding, Ministry of Agriculture and Rural Affairs, Hohhot, China

**Keywords:** Fleece Traits, Genetic Markers, Genome-wide Association Study, Inner Mongolia Cashmere Goats

## Abstract

**Objective:**

Genome-wide association study (GWAS) and haplotype analysis were employed to identify molecular markers and candidate genes associated with fleece traits in Inner Mongolia cashmere goats (IMCGs).

**Methods:**

GWASs using whole-genome resequencing data together with phenotypic data from 2,299 IMCGs, applying four models: mixed linear model, multiple locus mixed linear model, fixed and random model circulating probability unification, and Bayesian-information and linkage-disequilibrium iteratively nested keyway. We focused on the GWAS signals to conduct gene annotation and performed functional enrichment analyses to explore the biological processes underlying these signals. Additionally, haplotypes were constructed for the significant loci, and haplotype-phenotype association analyses were performed to identify molecular markers and candidate genes associated with these fleece traits in IMCGs.

**Results:**

We identified 542 SNPs and 179 candidate genes linked to fleece traits through GWAS and gene annotation. Genes such as *LAMA3*, *KCTD1*, *PTK7*, *FGFR3*, *LEF1*, *TAPT1*, *PTCH1*, *ELOVL6*, and *EVC* have emerged as important candidates that may influence fleece traits. Furthermore, 11 haplotype blocks related to fleece traits were constructed, among which A1A1, C1C1, E2E2, F1F1, G1G1, H1H1 and K1K1 were identified as the superior haplotype combinations for fleece traits. These could serve as important molecular markers to improve the accuracy of early selection and the economic efficiency of breeding programs for fleece traits in IMCGs.

**Conclusion:**

This study successfully employed GWAS to identify key genetic loci significantly associated with the fleece traits of IMCGs. The genetic basis of these traits was revealed through additional gene annotation and haplotype analysis. The findings provide important theoretical and practical foundations for molecular breeding in IMCGs.

## INTRODUCTION

The Inner Mongolia cashmere goats (IMCGs) is a superior indigenous breed developed through long-term natural and artificial selection. It is currently recognized as one of the most important dual-purpose breeds producing cashmere and meat [1]. Renowned for its fine cashmere diameter (CD), high cashmere yield (CY), and stable heritability, it is often used as an elite sire line for improving other cashmere goat breeds. Amidst the livestock industry’s transformation and rising consumer expectations, there is a growing need to further improve the fleece traits of IMCGs to meet current market requirements [2].

The concept of genome-wide association study (GWAS) was first proposed by Risch and Merikangas in 1996 [3]. This method is employed to identify genetic variations across the entire genome in multiple individuals and investigate associations between genetics and phenotypes at the population level. At present, GWAS has become a major tool for identifying candidate genes underlying complex quantitative traits in animals. The hair follicle, a structurally complex organ, has seen the identification of several candidate genes associated with its development to date, including *KRT85*, *KRT35*, *KRT2*7 [4], *FGF5*, *KRT71*, *TCHH*, *RSPO2* [5], *SGK3*, *IGFBP7*, *OXTR*, *ROCK1* [6], *KAP3-1*, *KRTAP8-1* and *KRTAP24-1* [7]. During hair follicle growth and development, each stage transition is regulated by distinct molecular mechanisms and signaling pathways, such as the Wnt, Hedgehog [8], Notch [9], TGF-β, EGF, FGF and BMP signaling pathways [10,11]. These pathways interact with each other, forming a complex regulatory network that orchestrates the hair follicle growth cycle.

To date, a series of chips have been developed, among which the commonly used SNP chips in goat research include the 10K liquid low-density cashmere goat chip [12], Illumina Caprine 50K BeadChip [13], Illumina 70 K Goat SNP chip [14]. These chips have become important tools for conducting GWAS on economically important traits in goats. While genotyping using chips for breeding purposes is relatively low-cost, it has limitations in terms of locus coverage, making it difficult to comprehensively capture genetic variations across the genome. This approach exhibits certain shortcomings, particularly in detecting low-frequency variants and novel mutations. Currently, although GWAS analyses for fleece traits in cashmere goats have been conducted, most are based on chip data or low-coverage sequencing data [2,12]. GWAS based on large-scale, high-depth whole-genome resequencing data has not yet been performed. It’s the first time to conduct GWAS for fleece traits in IMCGs with varying depth genome resequencing data and models.

The aim of this study was to identify causal variants and candidate genes significantly associated with fleece traits by conducting an integrated analysis of large-scale genomic data. Therefore, we utilized phenotypic records of fleece traits, systematic environmental effect data, resequencing data at varying depths (1X, 3X, 5X, and 10X) in IMCGs to perform the GWAS with mixed linear model (MLM), multiple locus mixed linear model (MLMM), fixed and random model circulating probability unification (FarmCPU), and Bayesian-information and linkage-disequilibrium iteratively nested keyway (BLINK) models. Moreover, haplotype block construction was performed for the identified significant loci, aiming to lay a foundation for the future molecular breeding of fleece traits in IMCGs.

## MATERIALS AND METHODS

### Animal population and phenotypic data sources

The phenotype data used in this study were obtained from the IMCGs (Arbas type) population maintained by Inner Mongolia Yiwei White Cashmere Goat. The traits in this study included CY, fiber length (FL), cashmere length (CL), and CD. A total of 30,429 phenotypic records were collected from individuals aged from 1 to 8 years. The average CY is 740.31 g with a standard deviation of 215.29 g, indicating a significant variation in yield among individuals. The average value of FL is 18.90 cm with a standard deviation of 4.90 cm. The average value of CL is 6.25 cm with a standard deviation of 1.10 cm, while the average value of CD is 15.23 μm with a standard deviation of 0.81 μm. The quality control of phenotype data was performed using R software [15], and outliers were removed with the criterion of mean±3 standard deviations. All traits were found to follow a normal distribution after quality control [16].

### Genotype data processing and quality control

This study utilized genotypic data from 2,299 IMCGs (Arbas type) with imputed sequencing depths of 1X, 3X, 5X, and 10X [16]. During data processing, the Seqtk software (ver. 1.4) was first used to perform random subsampling of the 10X whole-genome resequencing data from 471 individuals, thereby constructing reference datasets with sequencing depths of 1X, 3X, 5X, and 10X. Subsequently, genotype calling was performed by aligning the sequencing data to the Capra hircus reference genome assembled by our research group [17]. Then the genotype imputation was conducted using Beagle software (Ver. 5.4), the previously accumulated GGP_Goat_70K SNP chip genotype data from 2,299 IMCGs (Arbas type) were imputed into the 1X, 3X, 5X, and 10X resequencing datasets. To ensure data quality, strict quality control of the genotype data in each depth sequence was performed using PLINK software (ver. 1.9). The filtering criteria was as follow: minor allele frequency (MAF)<0.05, Hardy–Weinberg equilibrium p*-*value<1×10^−6^, and individual call rate<0.90. The retained SNPs were used for subsequent analyses.

### Genome-wide association study

To eliminate the effects of systematic environmental effects on phenotypic values, the adjusted phenotypic value were obtained using lme4 package in the R software (ver. 4.1). The model was specified as follows:


(1) $$y_{ijkl}=\mu +{\text{Year}}_{i}+{\text{Herd}}_{j}+{\text{Age}}_{k}+{\text{Sex}}_{l}+e_{ijkl}$$

(2) $$BLUE=y_{ijkl}-(\mu +{\text{Year}}_{i}+{\text{Herd}}_{j}+{\text{Age}}_{k}+{\text{Sex}}_{l})$$

where *y_ijkl_* is the observed phenotypic value of the i^th^ individual, *μ* is the overall mean of the trait, Year*_i_*, Herd*_j_*, Age*_k_* and Sex*_l_* denote the fixed effects of year, herd, individual age, and sex, respectively, and e is the residual error, BLUE is the adjusted phenotypic value.

In this study, GWAS were conducted for fleece traits in IMCGs using four distinct statistical models: MLM [18], MLMM [19], FarmCPU [20], and BLINK [21]. The analyses were performed using the GAPIT 3 package [22] in the R software.

The formula of MLM was as follows:


(3) y=Zβ+e

Where *y* is the phenotypic value of traits, *Z* is the random effect matrix, *β* is the vector of random effect, *β~N(0,Gσ_g_*^2^), *e* is the random error, *e*~*N*(0, δ*e*^2^).

The formula of MLMM was as follows:


(4) y=Zβ+Sd+Qv+e

Where *d* is the vector of false-positive quantitative trait nucleotide (QTN), *v* is the first three principal components (PCA) of individuals, *S* and *Q* are the structure matrix of false-positive QTN and first three PCA, respectively. The remaining parameters were consistent with those used in the MLM model.

The formula of FarmCPU was as follows:


(5) $$y=Z_{t}\beta_{t}+S_{i}d_{i}+e$$

(6) y=u+e

Where (5) is the fixed-effect model (FEM), (6) is the random-effect model (REM), and FarmCPU alternately runs FEM and REM. *Z_t_* is the incidence matrix of *β_t_*; *β* is the fixed effect vector of the QTN genotype matrix; t is the number of QTNs; *S_i_* represents the i^th^ SNP, and *d_i_* is the corresponding effect of the marker. *u* denotes the sum of the genetic effects of the experimental individuals.

The formula of BLINK was as follows:


(7) $$y_{i}=S_{i1}^{*}b_{1}+S_{i2}^{*}b_{2}+\ldots +S_{ik}^{*}b_{k}+S_{ij}d_{j}+e_{i}$$

(8) $$y_{i}=S_{i1}^{*}b_{1}+S_{i2}^{*}b_{2}+\ldots +S_{ik}^{*}b_{k}+e_{i}$$

(9) BIC=-2LL+2KLn(n)

The BLINK method involves two fixed effect models. In the formula (7), *y_i_* is the phenotype of the ith individual; *b*_1_, *b*_2_, …, *b_k_* are the vector of the kth pseudo QTNs effect; S_i_^*^_k_ is the structure matrix of in the i^th^ individual; *S_ij_* is the genotype of the *j^th^* SNP, *d_j_* is the effect of the *j^th^* SNP; *e_i_* is the residual. In the formula (8), based on the BIC, the best t pseudo QTNs are selected from the k pseudo QTNs, and the process continues until no new pseudo QTNs are identified or the maximum number of iterations is reached. In the formula (9), *LL* represents the log-likelihood value; *K* is the number of pseudo QTNs; *Ln* denotes the natural logarithm; and *n* is the number of individuals.

The Bonferroni correction method [23] was applied to determine the genome-wide significance threshold (adjusted p = 0.05/N, where N is the total number of SNPs). For 1X: p≈7.5×10^−7^; for 3X: p≈6.9×10^−7^; for 5X: p≈2.6×10^−7^; for 10X: p≈1.3×10^−7^. The CMplot package was applied to draw Manhattan and quantile-quantile (Q-Q) plots to visualize GWAS results.

### Gene Ontology and Kyoto Encyclopedia of Genes and Genomes enrichment analysis

Based on the genome annotation file (Goat.gff3.gz) from the Capra hircus reference genome assembled by our research group, gene annotation was performed using Bedtools [24]. Candidate genes were identified by extracting genomic regions spanning 50 kb upstream and downstream of significantly associated SNPs. These genes were subsequently annotated and preliminarily characterized as potential genetic markers influencing fleece traits in IMCGs. Gene Ontology (GO) and Kyoto Encyclopedia of Genes and Genomes (KEGG) pathway enrichment analyses were conducted using the DAVID website ( https://davidbioinformatics.nih.gov/). Multiple testing correction was performed using the Benjamini-Hochberg method, with a significance threshold set at p-value<0.1 and false discovery rate (FDR)<0.1.

### Haplotype construction and association analysis

Haplotypes were constructed for genome-wide significant SNPs that were predicted to be strongly associated with fleece traits, based on functional annotation and enrichment analysis performed using LDBlockShow software (ver. 1.40). Haplotype blocks were defined according to the method proposed by Gabriel et al [25], which identifies conserved regions with low historical recombination by calculating pairwise D’ values and confidence intervals between SNPs. Further association analyses were conducted between different haplotype combinations and the phenotypic values of fleece traits using SAS software (ver. 9.2), aiming to identify superior haplotypes and key functional variants linked to fleece traits.

## RESULTS

### Genotype quality control and principal component analysis

After quality control, the number of SNPs retained after quality control was 67,021, 72,018, 195,576, and 388,677, respectively The SNP density across chromosomes is shown in Supplement 1, indicating an even distribution of SNPs across all chromosomes. Linkage disequilibrium (LD) decay plots were generated for each sequencing depth. The results showed that LD decreased with increasing genetic or physical distance, and the decay rate gradually declined. Moreover, LD became more stable with increasing sequencing depth. PCA based on the different sequencing depths was performed to assess population structure (Supplement 2). No obvious stratification was observed among the 2,299 IMCGs. Therefore, there is no need to consider the impact of population structure in the subsequent GWAS analysis.

### Genome-wide association study for fleece traits in Inner Mongolia cashmere goats

**Figure 1:**
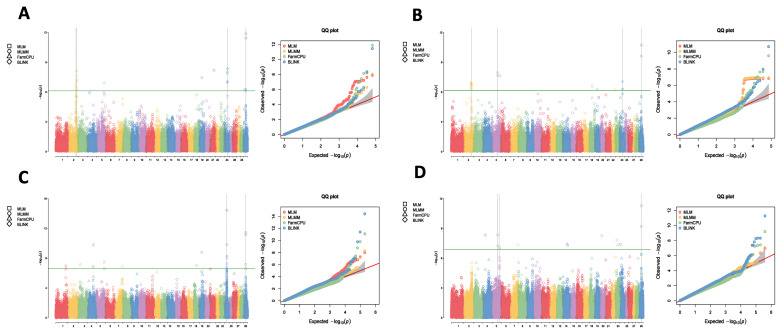
Manhattan plots and QQ plots for cashmere yield (CY) at varying sequencing depths across different GWAS models (MLM, MLMM, FarmCPU and BLINK). The green lines in the plots represent the genome-wide significance thresholds (0.05/n). (A) 1×_GWAS; (B) 3×_GWAS; (C) 5×_GWAS; (D) 10×_GWAS. QQ, quantile-quantile; MLM, mixed linear model; MLMM, multiple locus mixed linear model; FarmCPU, fixed and random model circulating probability unification; BLINK, Bayesian-information and linkage-disequilibrium iteratively nested keyway; GWAS, genome-wide association study.

**Figure 2:**
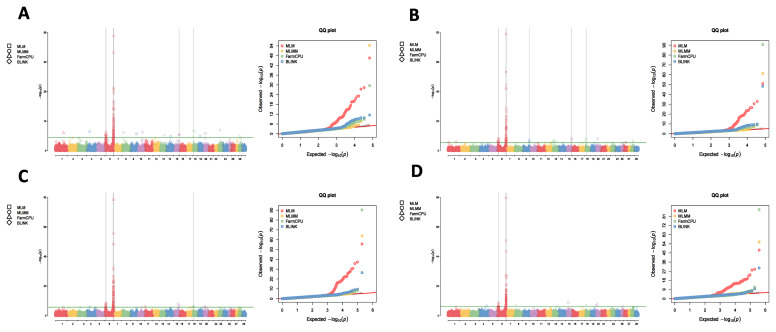
Manhattan plots and QQ plots for fiber length (FL) at varying sequencing depths across different GWAS models (MLM, MLMM, FarmCPU and BLINK). The green lines in the plots represent the genome-wide significance thresholds (0.05/n). (A) 1×_GWAS; (B) 3×_GWAS; (C) 5×_GWAS; (D) 10×_GWAS. QQ, quantile-quantile; MLM, mixed linear model; MLMM, multiple locus mixed linear model; FarmCPU, fixed and random model circulating probability unification; BLINK, Bayesian-information and linkage-disequilibrium iteratively nested keyway; GWAS, genome-wide association study.

**Figure 3:**
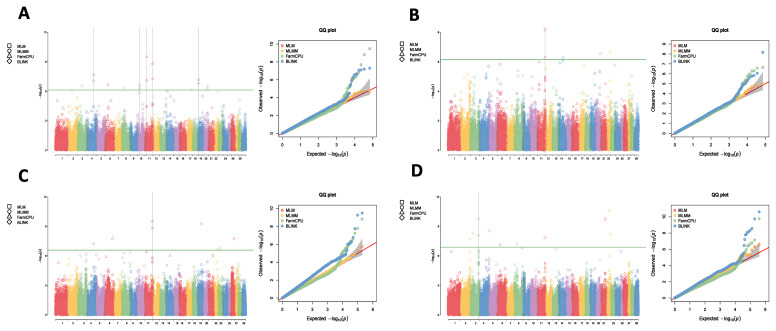
Manhattan plots and QQ plots for cashmere length (CL) at varying sequencing depths across different GWAS models (MLM, MLMM, FarmCPU and BLINK). The green lines in the plots represent the genome-wide significance thresholds (0.05/n). (A) 1×_GWAS; (B) 3×_GWAS; (C) 5×_GWAS; (D) 10×_GWAS. QQ, quantile-quantile; MLM, mixed linear model; MLMM, multiple locus mixed linear model; FarmCPU, fixed and random model circulating probability unification; BLINK, Bayesian-information and linkage-disequilibrium iteratively nested keyway; GWAS, genome-wide association study.

**Figure 4:**
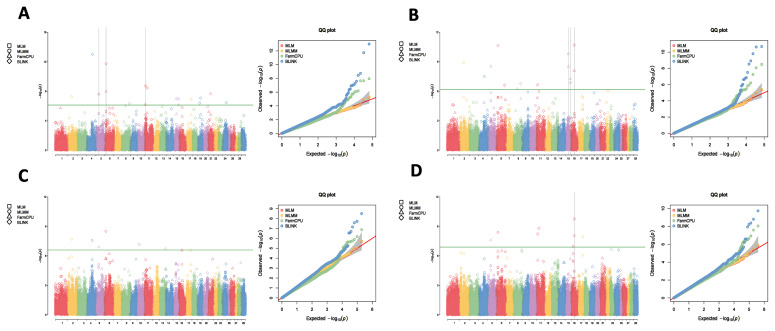
Manhattan plots and QQ plots for cashmere diameter (CD) at varying sequencing depths across different GWAS models (MLM, MLMM, FarmCPU and BLINK). The green lines in the plots represent the genome-wide significance thresholds (0.05/n). (A) 1×_GWAS; (B) 3×_GWAS; (C) 5×_GWAS; (D) 10×_GWAS. QQ, quantile-quantile; MLM, mixed linear model; MLMM, multiple locus mixed linear model; FarmCPU, fixed and random model circulating probability unification; BLINK, Bayesian-information and linkage-disequilibrium iteratively nested keyway; GWAS, genome-wide association study.

Utilizing the 1X, 3X, 5X, and 10X resequencing data from 2,299 IMCGs, we conducted GWAS using the MLM, MLMM, FarmCPU, and BLINK methods to detect the genetic markers that related to fleece traits in IMCGs. The results are presented in Figures 1–4. Significant SNPs associated with CY were identified on chromosomes 2, 5, 24, and 29, with a total of 62 significant SNPs detected across the four sequencing depths. For FL, significant SNPs were located on chromosomes 6, 15, and 29, with a total of 428 significant SNPs identified. Significant SNPs associated with CL were found on chromosomes 3, 4, 11, and 19, totalling 26 SNPs across the four sequencing depths. For CD, significant SNPs were identified on chromosomes 6, 11, 15, and 16, with a total of 26 significant SNPs detected.

### Functional annotation and enrichment analysis of candidate genes

Gene annotation was performed for the regions 50 kb upstream and downstream of the 62 SNPs significantly associated with CY, resulting in the identification of 49 candidate genes (Table 1). These included Serum Response Factor (*SRF*), Protein Tyrosine Kinase 7 (*PTK7*), Laminin Subunit Alpha 3 (*LAMA3*), and Tau Tubulin Kinase 1 (*TTBK1*) et al. Notably, Fanconi Anemia Complementation Group F (*FANCF*) and Growth Arrest-Specific 2 (*GAS2*) were consistently identified across all four sequencing depths (Table 2). For the 428 SNPs significantly associated with FL, a total of 76 candidate genes were annotated. These included Fibroblast Growth Factor Receptor 3 (*FGFR3*), Transmembrane Anterior Posterior Transformation 1 (*TAPT1*), and Regulator of G Protein Signaling 12 (*RGS12*) et al. Among them, 33 candidate genes were commonly identified across all four sequencing depths. The 26 SNPs significantly associated with CL was annotated 32 candidate genes, including Patched 1 (*PTCH1*), Hyaluronidase 4 (*HYAL4*), and HECT and RLD Domain Containing E3 Ubiquitin Protein Ligase 5 (*HERC5*) et al. For the 26 SNPs significantly associated with CD, a total of 22 candidate genes were identified. Among them, ELOVL Fatty Acid Elongase 6 (*ELOVL6*) was consistently detected across all sequencing depths.

**Figure 5:**
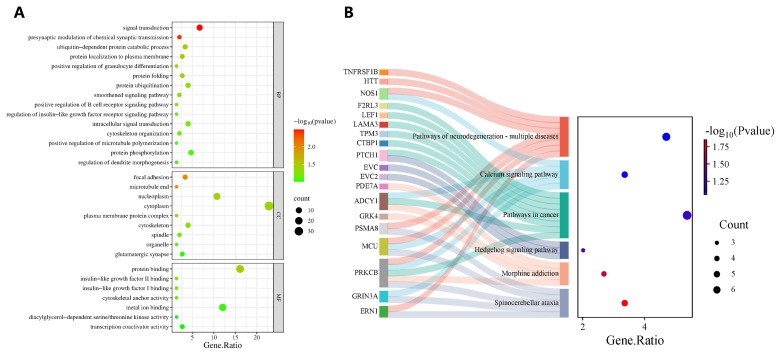
GO and KEGG enrichment analysis of fleece traits of IMCGs. (A) GO enrichment analysis of fleece traits; significantly enriched terms defined by p<0.1. (B) KEGG pathway enrichment analysis of fleece traits; significantly enriched pathways defined by p<0.1. GO, Gene Ontology; KEGG, Kyoto Encyclopedia of Genes and Genomes; IMCGs, Inner Mongolia cashmere goats.

A total of 179 candidate genes significantly associated with fleece traits were subjected to GO and KEGG enrichment analyses. The results are shown in Figure 5. GO enrichment analysis revealed that these genes are potentially involved in several key biological processes, including signal transduction (GO:0007165), presynaptic regulation of chemical synaptic transmission (GO:0099171), focal adhesion (GO:0005925), and ubiquitin-dependent protein catabolic process (GO: 0006511). KEGG pathway analysis indicated that these genes were most enriched in the Hedgehog signaling pathway, calcium signaling pathway, spinocerebellar ataxia, morphine addiction, and various cancer-related pathways. Notably, *PTCH1* and *EVC* were enriched in the Hedgehog signaling pathway, which is closely associated with hair follicle development, cell cycle regulation, and hair growth.

### Linkage disequilibrium analysis and haplotype block construction

**Figure 6:**
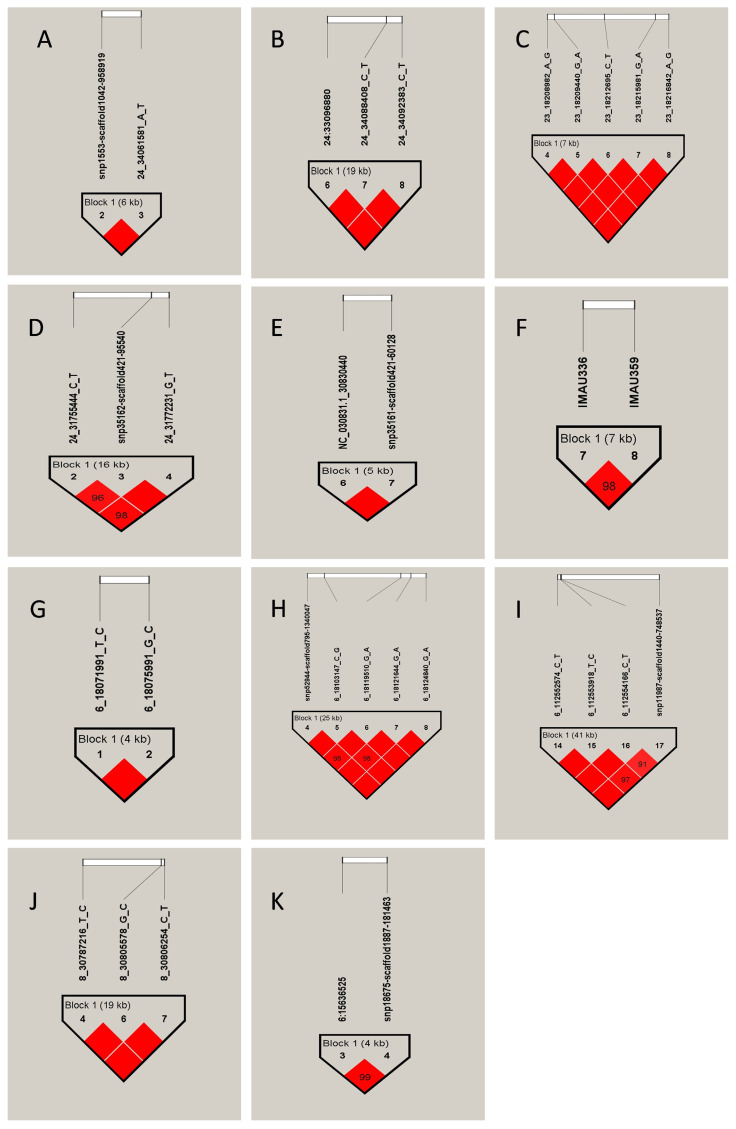
Results of linkage disequilibrium analysis and haplotype block of significantly associated SNPs of fleece traits in IMCGs. (A–K) Represent 11 haplotypes, respectively. IMCGs, Inner Mongolia cashmere goats.

LD analysis was performed for the loci corresponding to genes enriched in key pathways. The results are shown in Table 3 and Figure 6. The candidate genes *LAMA3*, *PTK7*, and *KCTD1* were significantly associated with CY, and five haplotype blocks were constructed within the 50 kb upstream and downstream regions of the corresponding significant SNPs. For FL, the candidate genes *FGFR3*, *LEF1*, and *TAPT1* were identified, and four haplotype blocks were constructed based on the ±50 kb regions around the associated SNPs. For CL, the *PTCH1* was identified to be as the key gene. And one haplotype block was constructed. For CD, the candidate genes *ELOVL6* and *EVC* were identified. One haplotype block was constructed around the ±50 kb region of the significant SNP corresponding to *ELOVL6*. However, no haplotype block was formed within the ±50 kb region surrounding the significant SNP associated with *EVC*.

### Association analysis of haplotype combination and phenotypic value of fleece traits

**Figure 7:**
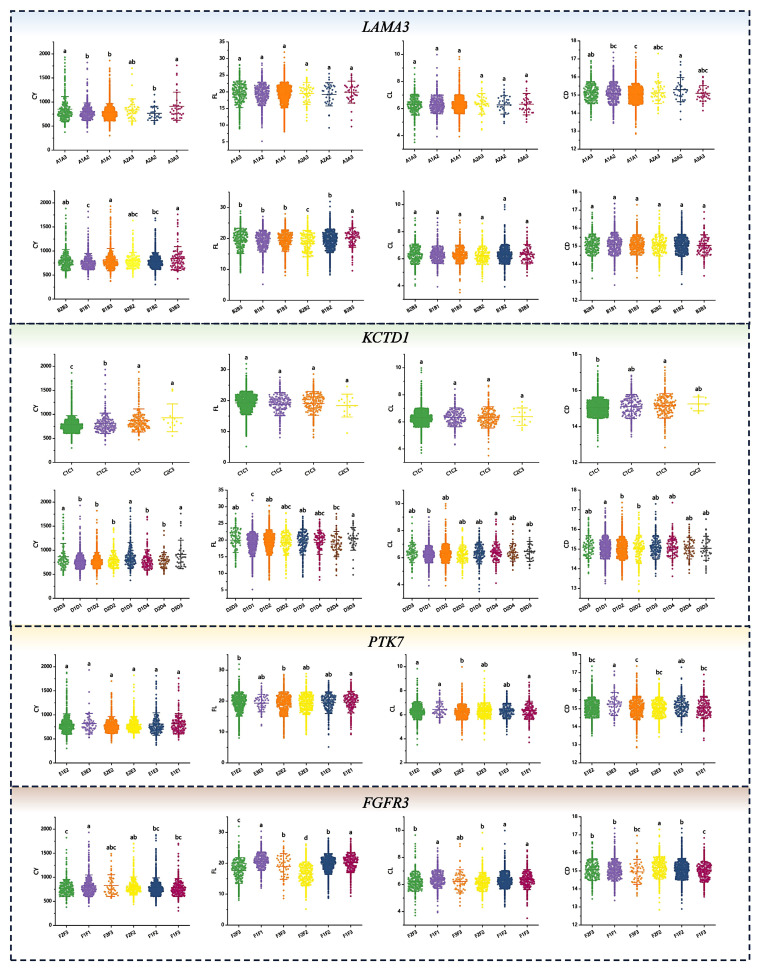
Association analysis between haplotype combinations and fleece traits. Each row represents a haplotype combination, with columns from left to right corresponding to cashmere yield (CY), fiber length (FL), cashmere length (CL), and cashmere diameter (CD). *x*-axis indicates the haplotype combinations, and *y*-axis represents the phenotypic values associated with fleece traits. ^a–c^ Different letters indicate significant differences, while the same letters indicate no significant difference (p<0.05).

**Figure 8:**
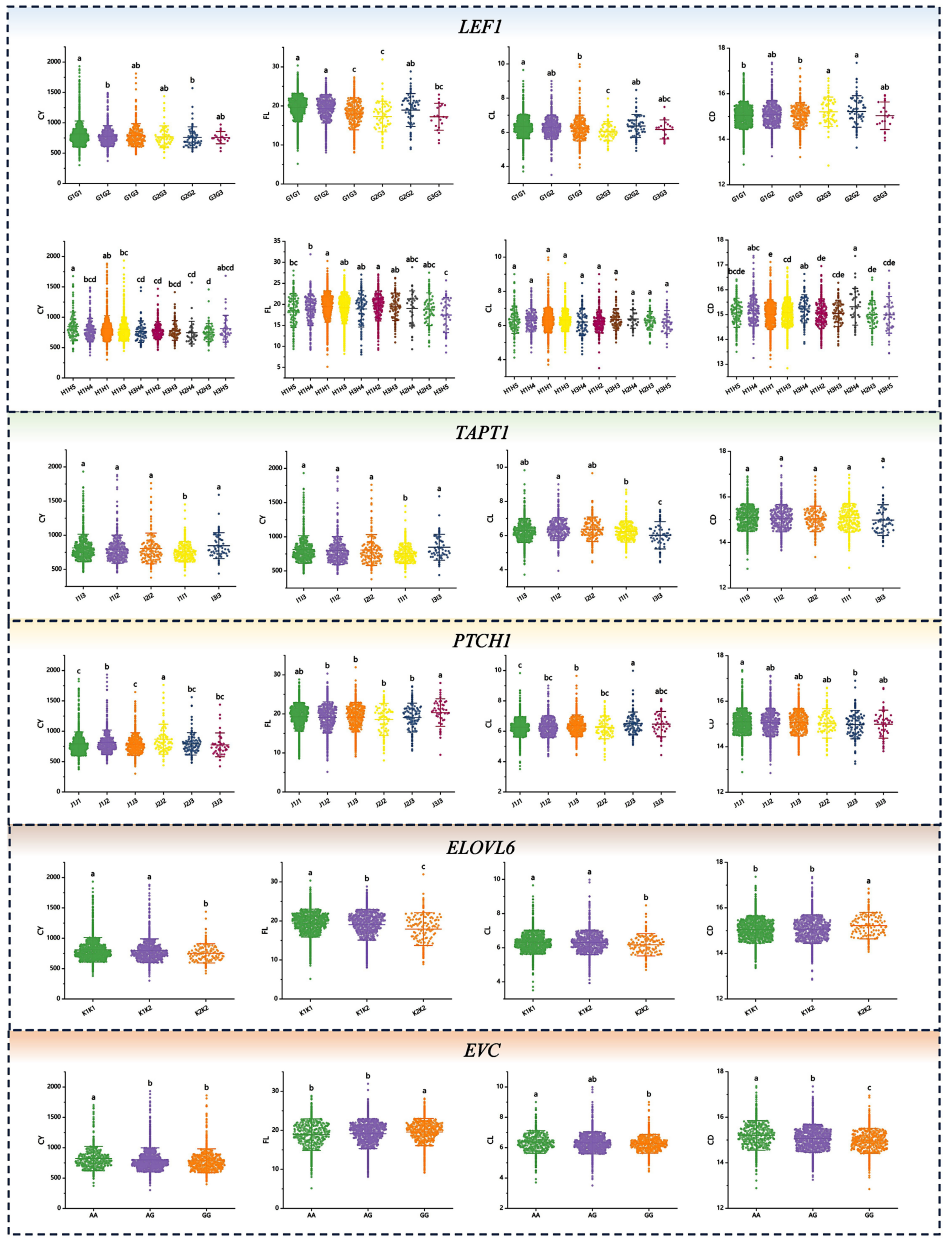
Association analysis between haplotype combinations and fleece traits. Each row represents a haplotype combination, with columns from left to right corresponding to cashmere yield (CY), fiber length (FL), cashmere length (CL), and cashmere diameter (CD). *x*-axis indicates the haplotype combinations, and *y*-axis represents the phenotypic values associated with fleece traits. ^a–e^ Different letters indicate significant differences, while the same letters indicate no significant difference (p<0.05).

Association analyses were conducted between haplotype blocks and the corresponding phenotypic values of each fleece trait. The results are shown in Supplement 3 and Figures 7, 8. Significant associations between haplotype combinations and fleece traits were identified in IMCGs (p<0.05). For CY, superior haplotype combinations included A1A3, A3A3, B1B3, B3B3, C1C3, C2C3, D1D3, D2D3, D3D3, F1F1, G1G1, H1H5, I1I2, I1I3, I2I2, I3I3, J2J2, K1K1, and K1K2. The phenotypic value of FL was significantly associated with B3B3, D3D3, E1E1, F1F1, F1F3, G1G1, G1G2, H1H1, H1H2, I1I2, I2I2, J3J3, and K1K1 combinations. For CL, the phenotypic values with D1D4, E1E1, E1E2, E3E3, F1F1, F1F3, G1G1, I1I2, J2J3, K1K1, and K1K2 showed significantly higher. Conversely, for CD, A1A1, C1C1, D1D1, D2D2, E2E2, F1F3, G1G1, G1G3, H1H1, J2J3, K1K1, and K1K2 exhibited significantly lower phenotypic values. At SNP-6:104712402 (A>G), individuals with the AA genotype showed significantly higher phenotypic values than those with AG and GG genotypes for CY and CL (p<0.05). For FL, individuals with the GG genotype had significantly higher phenotypic values compared to that with AA and AG genotypes (p<0.05). Conversely, for CD, phenotypic values with GG were significantly lower than that with AA and AG (p<0.05).

## DISCUSSION

With the rapid development of genomic technologies, GWAS have become an essential approach for uncovering the genetic architecture of complex traits. In livestock breeding, GWAS plays a critical role in identifying loci associated with economically important traits. This study conducted GWAS by analyzing genotypic data obtained at varying sequencing depths (1X, 3X, 5X, and 10X) using four distinct statistical models (MLM, MLMM, FarmCPU, and BLINK). A total of 49 genes were identified to be significantly associated with CY. Among them, the *LAMA3* belongs to the laminin family and responds to a variety of epithelial-mesenchymal regulatory factors, such as keratinocyte growth factor, epidermal growth factor, and skin fibrosis mediators. Previous studies have demonstrated that *LAMA3* point mutations in male mice lead to a significant proliferation of sebaceous glands in the dermal layer, concomitant with structural degeneration of hair follicles [26]. The Wnt signaling pathway is one of the key pathways regulating hair follicle morphogenesis and cycling. It was also the first signaling pathway discovered to promote hair follicle development by regulating the formation of the basement membrane [27]. The canonical Wnt signaling pathway involves multiple components, including Wnt proteins, the cell surface Frizzled (FZD) receptor family, Dishevelled (DSH) proteins, β-catenin, and the axin/GSK-3/APC complex [28]. Previous studies have demonstrated that *KCTD1*-encoded protein interacts with β-catenin, facilitating its sequential phosphorylation by CK1 and GSK-3β, ultimately leads to β-catenin degradation via the β-TrCP-mediated ubiquitin-proteasome pathway. This process ultimately inhibits the activity of the canonical Wnt/β-catenin signaling pathway [29]. Additionally, the protein encoded by the *PTK7* is a transmembrane receptor that has been confirmed to play an essential role in the activation of canonical Wnt signal transduction [30]. In this study, the *PTK7* was significantly enriched in metal ion binding and protein phosphorylation, whereas *LAMA3* was associated with cancer-related pathways. Metal ions are crucial for growth and development of goat, playing roles in protection transport, signaling, and waste excretion.

For the FL, 76 significantly associated genes were annotated. Previous studies have demonstrated that the Wnt/β-catenin signaling pathway serves as a critical regulator during early hair follicle development, orchestrating hair follicle morphogenesis. Notably, this pathway represents one of the primary signaling cascades that direct epidermal cell differentiation into hair follicle structures [31]. Among the key components, the *LEF1* encodes a transcription factor that plays a central role in the Wnt/β-catenin pathway. It regulates gene expression in dermal papilla cells, thereby driving hair follicle differentiation and cyclical growth [32]. Previous studies have shown that fibroblast growth factor (FGF) signaling may play an important role in modulating the hair growth cycle [33]. A comprehensive analysis of mRNA expression patterns for all 22 FGF family members and their four receptors (FGFRs) in murine full-thickness skin revealed that *FGFR3* expression reached its peak during the 18^th^ day of the hair cycle [34]. Furthermore, *TAPT1* encodes a highly conserved protein, and studies have shown that mutations in this gene may impair primary cilia formation in zebrafish [35].

Through annotation of 26 significant SNPs associated with CL, we identified 32 candidate genes. Notably, the Sonic hedgehog (SHH) signaling pathway, known to function downstream of Wnt signalling, has been well-established as a crucial regulator of hair follicle proliferation and maintenance. Among these genes, *PTCH1* encodes the core inhibitory receptor of the Hedgehog (Hh) signaling pathway, which negatively regulates Hh signaling by suppressing the activity of Smoothened (SMO) protein, thereby contributing to the phenotypic homeostasis of hair follicle stem cells [36]. Meanwhile, *PTCH1* was significantly enriched in GO terms, such as signal transduction, SMO signaling pathway, and Hh signaling pathway. These findings suggest that *PTCH1* may play a critical role in modulating the hair growth cycle.

Through comprehensive annotation of 26 SNPs associated with CD, we identified 22 putative candidate genes. Among these, *ELOVL6* serves as an important paralog of *ELOVL3*, with both genes involved in the elongation of long-chain fatty acids. In previous research on Southern Xinjiang cashmere goats [37], experimentally validated the differentially expressed gene *ELOVL3*, demonstrating its role in promoting the proliferation of dermal papilla cells in secondary hair follicles, suggesting its potential function in follicular development. Another noteworthy candidate gene, *EVC*, along with *EVC2*, acts as an essential positive regulator of the Hh signaling pathway [38]. These genes functionally cooperate with SMO to assemble active signaling complexes, thereby enabling robust downstream signal transduction [39]. In this study, *EVC* showed significant enrichment in multiple functional annotation terms, including signal transduction, cytoskeleton, and Hh signaling pathway.

Haplotypes refer to combinations of closely linked allelic markers located on the same chromosome that are usually inherited together in a coordinated manner [25]. Haplotype analysis has emerged as a powerful approach in GWAS, enabling both the precise localization of heterogeneous SNPs and the identification of candidate genes. By capturing the combined effects and epistatic interactions of multiple SNPs, haplotype-based methods offer superior statistical power compared to single-SNP analyses for dissecting the genetic architecture of complex traits [40]. In this study, LD analysis was conducted for the genes discussed above, resulting in the identification of 11 haplotype blocks. Haplotype-phenotype association analysis revealed significant associations for 19, 13, 12, and 12 haplotype combinations significantly associated with fleece traits. We focused on homozygous haplotypes with higher frequencies in high-performing groups, deeming them superior for each trait. Specifically, F1F1, G1G1, and K1K1 were identified as superior haplotype combinations for CY, F1F1, G1G1, H1H1, and K1K1 for FL, G1G1 and K1K1 for CL, and A1A1, C1C1, E2E2, G1G1, H1H1, and K1K1 for CD. Notably, G1G1 and K1K1 were identified as superior haplotype combinations across all fleece traits, indicating their potential role in main effect QTL regions or regulatory genes. This insight into trait-specific haplotypes could uncover new biological pathways. For the SNP site SNP-6:104712402 A>G corresponding to the *EVC*, no haplotype block formed around the significant SNP due to the low number and sparse distribution of SNPs in the region. The wild-type genotype has a positive regulatory effect on CY and CL, while the wild-type genotype may lead to coarser fiber, thereby affecting fleece quality. The observed inconsistency in trait correlations suggests potential antagonistic pleiotropy at this locus. Consequently, breeding strategies should incorporate genetic trade-offs between traits to mitigate unintended negative consequences of single-trait selection.

## CONCLUSION

Based on GWAS and functional annotation, this study systematically identified candidate genes and potential functional variants associated with fleece traits in IMCGs. The total of 542 SNPs and 179 candidate genes linked to fleece traits were identified. The *LAMA3*, *KCTD1, PTK7*, *FGFR3*, *LEF1*, *TAPT1*, *PTCH1*, *ELOVL6*, and *EVC* were identified as important candidates potentially influencing fleece traits. LD analysis performed within the candidate regions further identified 11 haplotype blocks associated with fleece traits. Among them, haplotypes A1A1, C1C1, E2E2, F1F1, G1G1, H1H1, and K1K1 were identified as superior haplotype combinations for fleece traits and may serve as important molecular markers for breeding applications. This will lay an important foundation for further investigating the molecular genetic mechanisms underlying the fleece traits of IMCGs.

## Figures and Tables

**Table 1:** Candidate genes significantly associated with fleece traits at different sequencing depths

Trait	Depth	Number of SNP	Number of gene	Candidate gene
CY	1X	17	13	*S19A3, TENS1, MYADM, LPIN2, EMIL2, FANCF, SAC31, BATF2, ARL2, SNX15, NALDL, CDCA5, GAS2*
	3X	29	9	*PRS58, RL37P, IBP5, IBP2, SMAL1, LAMA3, FANCF, GAS2, LARG1*
	5X	13	16	*PSF3, PSMA8, SSXT, KCTD1, TAF4B, FANCF, GAS2, WDR3, GDAP2, SPG17, ST17A, COA1, HECW1, PAR4, SIN3B, CPMD8*
	10X	11	17	*DNPH1, TF2H4, SRF, SYVM, DDR1, MUC21, CUL9, TTBK1, PTK7, CD226, DOK6, FANCF, GAS2, APOL3, ETV6, CALR3, EP15R*
FL	1X	113	54	*SPRE1, CLVS1, CK049, UBP10, RCAN2, MCU, PCLO, MAGAA, PPIA, SORC2, ADA2C, TM271, NKX11, SPON2, PITX2, HMX1, HGFA, GRK4, 3BP2, HAUS3, MFS10, MAEA, FGFR3, AMRP, NOP14, UVSSA, CBPZ, LETM1, CTBP1, DPOLN, DOK7, TNIP2, CPLX1, PDE6B, TMD11, HTRA3, TRM44, RNF4, CFA99, PIGG, ADDA, S3TC1, PCGF3, ACOX3, AFAP1, LEF1, RGS12, HD, F193A, ABLM2, SYNP2, KCC2D, NMD3A, TET5A*
	3X	65	50	*CK049, UBP10, CP17A, WBP1L, MAGAA, PPIA, SORC2, ADA2C, TM271, NKX11, SPON2, PITX2, HMX1, GRK4, MAD4, HAUS3, MFS10, MAEA, FGFR3, AMRP, NOP14, UVSSA, CBPZ, LETM1, CTBP1, DPOLN, DOK7, CPLX1, PDE6B, TMD11, HTRA3, TRM44, RNF4, CFA99, PIGG, ADDA, S3TC1, PCGF3, ACOX3, LST2, TAPT1, K0232, AFAP1, TBC14, RGS12, HD, F193A, ABLM2, SYNP2, ERAP1*
	5X	99	58	*TNIK, CK049, D42E1, UBP10, DHB2, MAGAA, PPIA, SORC2, ADA2C, TM271, NKX11, SPON2, CCD96, PITX2, HMX1, HGFA, GRK4, 3BP2, MAD4, HAUS3, MFS10, MAEA, FGFR3, AMRP, NOP14, TAD2B, GRPE1, UVSSA, CBPZ, LETM1, CTBP1, DPOLN, DOK7, TNIP2, CPLX1, PDE6B, TMD11, HTRA3, TRM44, RNF4, CFA99, PIGG, ADDA, S3TC1, PCGF3, ACOX3, LST2, TAPT1, CPEB2, K0232, AFAP1, TBC14, RGS12, C1QT7, HD, F193A, SYNP2, LDB2*
	10X	282	43	*TECTA, ZN469, FOG1, SNX18, MAGAA, PPIA, SORC2, ADA2C, TM271, NKX11, SPON2, PITX2, HMX1, GRK4, MSD1, MAD4, HAUS3, MFS10, AMRP, NOP14, UVSSA, CBPZ, CTBP1, DPOLN, DOK7, CPLX1, PDE6B, TMD11, HTRA3, TRM44, CFA99, PIGG, ADDA, S3TC1, PCGF3, ACOX3, LST2, AFAP1, RGS12, HD, ABLM2, LDB2, NMD3A*
CL	1X	11	13	*CC50B, KPCL, ATLA2, FBX21, CHP3, NOS1, ANKF1, HYAL4, PREY, HERC5, HERC6, FGD3, BICD2*
	3X	4	6	*TPM3, MTFR1, PDE7A, GORS1, TT21A, WDR48*
	5X	7	10	*ICAM2, ERN1, GORS1, TT21A, WDR48, STALP, ANR22, HYAL4, PPR3F, RHG24*
	10X	10	10	*VW5B1, KLH25, RBMS3, ANM6, CA094, NAV3, PREY, HERC5, HERC6, PTCH1*
CD	1X	14	9	*ELOVL6, ILRL2, ADCY1, TTC7A, SPNS2, SPNS3, PHB, SIA8E, LOXH1*
	3X	11	9	*EMX1, SFXN5, TNR1B, TNR8, TEX47, EVC, ELOVL6, LBN, CNTP3*
	5X	6	4	*PKCB1, EYA2, TEX47, ELOVL6*
	10X	7	4	*ZN638, SRBD1, DYSF, ELOVL6*

CY, cashmere yield; FL, fiber length; CL, cashmere length; CD, cashmere diameter.

**Table 2:** Common candidate genes of different sequencing depths for fleece traits

Trait	Depth	Number of common gene	Gene
CY	1X, 3X, 5X, 10X	2	*FANCF, GAS2*
FL	5X, 10X	1	*LDB2*
	3X, 5X	3	*TAPT1, K0232, TBC14*
	1X, 10X	1	*NMD3A*
	1X, 5X	3	*HGFA, 3BP2, TNIP2*
	3X, 5X, 10X	2	*MAD4, LST2*
	1X, 3X, 10X	1	*ABLM2*
	1X, 3X, 5X	8	*CK049, UBP10, MAEA, FGFR3, LETM1, RNF4, F193A, SYNP2*
	1X, 3X, 5X, 10X	33	*MAGAA, PPIA, SORC2, ADA2C, TM271, NKX11, SPON2, PITX2, HMX1, GRK4, HAUS3, MFS10, AMRP, NOP14, UVSSA, CBPZ, CTBP1, DPOLN, DOK7, CPLX1, PDE6B, TMD11, HTRA3, TRM44, CFA99, PIGG, ADDA, S3TC1, PCGF3, ACOX3, AFAP1, RGS12, HD*
CL	3X, 5X	3	*GORS1, TT21A, WDR48*
	1X, 10X	3	*PREY, HERC5, HERC6*
	1X, 5X	1	*HYAL4*
CD	3X, 5X	1	*TEX47*
	1X, 3X, 5X, 10X	1	*ELOVL6*

CY, cashmere yield; FL, fiber length; CL, cashmere length; CD, cashmere diameter.

**Table 3:** Haplotype analysis of SNPs linked to fleece traits in IMCGs

Traits	Gene	Number of SNP	Tag	Haplotype	Frequency
CY	*LAMA3*	24:34055176, 24:34061581	A1	TA	0.776
			A2	GA	0.121
			A3	TT	0.103
		24:34073122, 24:34088408, 24:34092383	B1	TCC	0.445
			B2	ACC	0.327
			B3	TTT	0.227
	*PTK7*	23:18208982,23:18209440,23:18212695, 23:18215981,23:18216842	C1	AGCGA	0.883
			C2	AGCGG	0.059
			C3	GATAG	0.058
	*KCTD1*	24:31755444, 24:31769167, 24:31772231	D1	CTG	0.560
			D2	CAG	0.289
			D3	TTT	0.098
			D4	CTT	0.052
		24:31798614, 24:31803824	E1	GA	0.296
			E2	TA	0.526
			E3	GG	0.178
FL	*FGFR3*	6:118040198, 6:118047593	F1	GG	0.438
			F2	TA	0.395
			F3	GA	0.165
	*LEF1*	6:18071991, 6:18075991	G1	TG	0.729
			G2	CG	0.176
			G3	TC	0.095
		6:18099504, 6:18103147, 6:18119510, 6:18121644, 6:18124840	H1	ACGGG	0.567
			H2	GCAGG	0.086
			H3	GCGGG	0.202
			H4	AGGAG	0.085
			H5	ACGGA	0.051
	*TAPT1*	6:112552574, 6:112553918,6:112554166,6:112594424	I1	CCCT	0.350
			I2	CCTT	0.267
			I3	CCCG	0.201
CL	*PTCH1*	8:30787216, 8:30805578, 8:30806254	J1	CCT	0.661
			J2	CGC	0.190
			J3	TGC	0.149
CD	*ELOVL6*	6:16239596, 6:16244037	K1	TA	0.742
			K2	GG	0.256
	*EVC*	SNP-6:104712402A>G	AA, AG, GG		-

Haplotypes sorted according to the 26 letters of the alphabet.

IMCGs, Inner Mongolia cashmere goats; CY, cashmere yield; FL, fiber length; CL, cashmere length; CD, cashmere diameter.

## Data Availability

Upon reasonable request, the datasets of this study can be available from the corresponding author.

## SUPPLEMENTARY MATERIAL

**Supplement 1**. Chromosome-specific SNP density within 1 Mb genomic intervals and the attenuation of LD in Inner Mongolia Cashmere Goats.

**Supplement 2**. Principal component analysis plots of population structure.

**Supplement 3**. Association analysis between haplotype combinations and fleece traits.
