# Supplementary material for: Genome-wide association study dissection of candidate genes for fleece traits in Inner Mongolia cashmere goats based on whole-genome resequencing data
Source: Anim Biosci. 2025 Dec 18;39(5):250631. doi: 10.5713/ab.250631 (PMC13175068; doi:10.5713/ab.250631)
Supplement: Supplementary file 1 [file ab-250631-Suppplement-1.pdf]

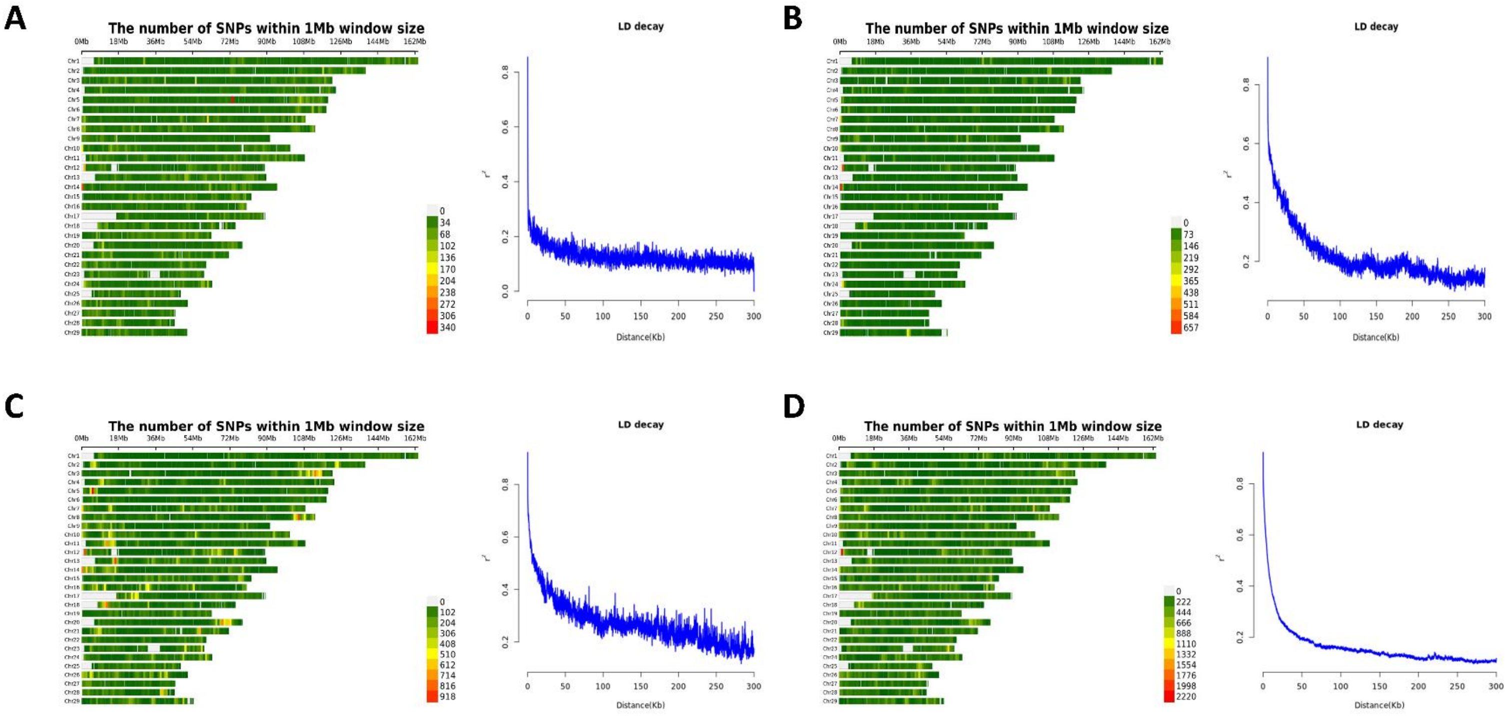

Supplement 1. Chromosome-specific SNP density within 1 Mb genomic intervals and the attenuation of LD in Inner Mongolia Cashmere Goats. (A)1X; (B) 3X; (C) 5X; (D) 10X.
