# Supplementary figures and images for: Genome-wide association study dissection of candidate genes for fleece traits in Inner Mongolia cashmere goats based on whole-genome resequencing data
Source: Anim Biosci. 2025 Dec 18;39(5):250631. doi: 10.5713/ab.250631 (PMC13175068; doi:10.5713/ab.250631)

**A**

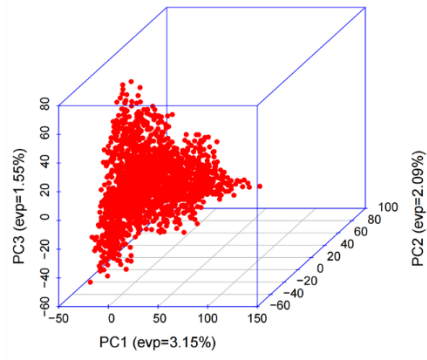

**B**

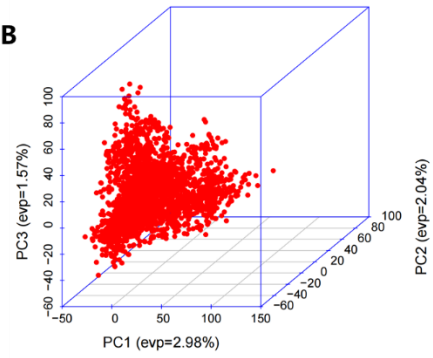

**C**

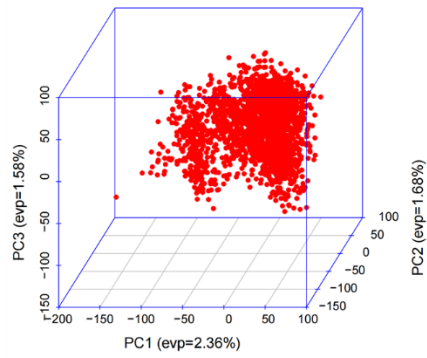

**D**

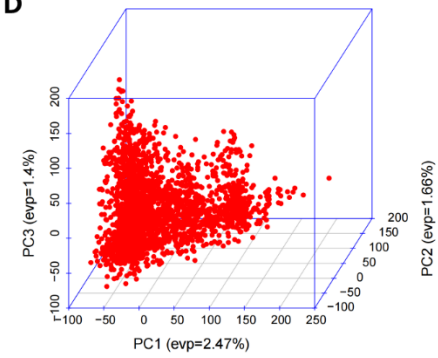

4

5 **Supplement 2.** Principal component analysis plots of population

6 structure. (A)1X; (B) 3X; (C) 5X; (D) 10X.

Supplement: Supplementary file 2 [file ab-250631-Suppplement-2.pdf]
