# Supplementary material for: Genome-wide association study dissection of candidate genes for fleece traits in Inner Mongolia cashmere goats based on whole-genome resequencing data
Source: Anim Biosci. 2025 Dec 18;39(5):250631. doi: 10.5713/ab.250631 (PMC13175068; doi:10.5713/ab.250631)
Supplement: Supplementary file 3 [file ab-250631-Suppplement-3.pdf]

Supplement 3. Association analysis between haplotype combinations and fleece traits.

Haplotype combination	Number	CY	Number	FL	Number	CL	Number	CD
A1A3(TTTA)	297	850.35±261.12[a]	328	19.26±4.00[a]	328	6.29±0.78[a]	320	15.13±0.61[ab]
A1A2(GTAA)	399	795.99±184.29[b]	421	19.37±3.56[a]	421	6.31±0.69[a]	417	15.09±0.65[bc]
A1A1(TTAA)	1309	786.61±178.34[b]	1367	19.06±3.83[a]	1367	6.32±0.69[a]	1345	15.04±0.60[c]
A2A3(GTTA)	48	835.04±231.38[ab]	51	19.49±3.32[a]	51	6.34±0.76[a]	51	15.13±0.60[abc]
A2A2(GGAA)	35	765.23±148.35[b]	37	19.24±3.48[a]	37	6.27±0.68[a]	36	15.30±0.67[a]
A3A3(TTTT)	37	912.65±291.74[a]	41	19.91±3.28[a]	41	6.32±0.79[a]	40	15.10±0.43[abc]
B2B3(ATTCTC)	314	809.02±219.85[ab]	329	19.22±4.09[b]	329	6.34±0.75[a]	326	15.06±0.61[a]
B1B1(TTCCCC)	408	777.34±170.69[c]	430	19.15±3.54[b]	430	6.29±0.66[a]	426	15.11±0.66[a]
B1B3(TTTCTC)	442	819.99±231.30[a]	467	19.26±3.52[b]	467	6.31±0.69[a]	454	15.07±0.57[a]
B2B2(AACCCC)	213	798.58±171.82[abc]	223	18.33±4.25[c]	223	6.26±0.69[a]	219	15.06±0.61[a]
B1B2(ATCCCC)	639	790.58±173.98[bc]	677	19.21±3.83[b]	677	6.33±0.73[a]	667	15.04±0.62[a]
B3B3(TTTTTT)	104	840.63±247.85[a]	114	20.25±3.21[a]	114	6.34±0.72[a]	112	15.05±0.59[a]
C1C1(AAGGCCGGAA)	1786	787.52±184.10[c]	1745	19.26±3.77[a]	1745	6.31±0.70[a]	1720	15.05±0.59[b]
C1C2(AAGGCCGGGA)	243	814.83±212.15[b]	235	18.85±3.75[a]	235	6.33±0.70[a]	232	15.12±0.67[ab]
C1C3(GAAGTCAGGA)	241	871.44±240.49[a]	237	19.11±3.83[a]	237	6.33±0.79[a]	230	15.16±0.69[a]
C2C3(GAAGTCAGGG)	12	928.84±288.71[a]	13	18.40±3.70[a]	13	6.38±0.65[a]	8	15.26±0.38[ab]
D2D3(TCATTG)	110	864.89±277.40[a]	120	19.97±3.71[ab]	120	6.39±0.76[ab]	118	15.10±0.58[ab]
D1D1(CCTTGG)	666	774.10±169.55[b]	698	18.80±3.76[c]	698	6.26±0.64[b]	686	15.10±0.61[a]
D1D2(CCATGG)	734	784.71±167.64[b]	768	19.24±3.83[ab]	768	6.32±0.73[ab]	757	15.03±0.61[b]

D2D2(CCAAGG)	163	805.90±188.78^{b}	173	19.21±3.77^{abc}	173	6.27±0.67^{ab}	171	15.00±0.71^{b}
D1D3(TCTTTG)	194	892.38±272.01^{a}	212	19.41±3.88^{ab}	212	6.35±0.81^{ab}	208	15.09±0.61^{ab}
D1D4(CCTTTG)	137	796.35±212.43^{b}	139	19.34±3.62^{abc}	139	6.41±0.77^{a}	138	15.10±0.56^{ab}
D2D4(CCATTG)	57	780.61±166.79^{b}	58	18.79±3.81^{bc}	58	6.40±0.65^{ab}	58	15.05±0.55^{ab}
D3D3(TTTTTT)	34	913.36±296.23^{a}	41	20.37±3.40^{a}	41	6.47±0.74^{ab}	40	15.04±0.61^{ab}
E1E2(GTAA)	673	807.06±213.61^{a}	706	19.03±3.91^{b}	706	6.34±0.76^{a}	698	15.07±0.58^{bc}
E3E3(GGGG)	66	816.06±213.87^{a}	69	19.34±2.84^{ab}	69	6.45±0.68^{a}	68	15.26±0.64^{a}
E2E2(TTAA)	574	786.67±175.84^{a}	607	18.99±3.98^{b}	607	6.24±0.67^{b}	597	15.04±0.65^{c}
E2E3(GTAG)	418	795.09±169.16^{a}	439	19.28±3.67^{ab}	439	6.32±0.69^{ab}	433	15.04±0.60^{bc}
E1E3(GGAG)	218	808.61±233.13^{a}	228	19.45±3.51^{ab}	228	6.31±0.65^{ab}	224	15.14±0.58^{ab}
E1E1(GGAA)	176	814.05±212.45^{a}	196	19.66±3.51^{a}	196	6.37±0.76^{a}	189	15.07±0.62^{bc}
F2F3(GTAA)	309	776.18±178.21^{c}	321	17.62±4.18^{c}	321	6.24±0.74^{b}	314	15.07±0.60^{b}
F1F1(GGGG)	402	821.80±226.92^{a}	342	20.65±2.99^{a}	432	6.40±0.69^{a}	426	15.06±0.62^{b}
F3F3(GGAA)	53	828.18±225.57^{abc}	60	18.94±4.14^{b}	60	6.23±0.86^{ab}	57	14.95±0.70^{bc}
F2F2(TTAA)	321	817.76±189.66^{ab}	325	16.53±3.85^{d}	325	6.18±0.71^{b}	321	15.17±0.63^{a}
F1F2(GTGA)	756	794.00±192.76^{bc}	802	19.73±3.34^{b}	802	6.34±0.67^{a}	787	15.09±0.60^{b}
F1F3(GGGA)	281	786.32±187.85^{bc}	302	20.21±3.27^{a}	302	6.36±0.74^{a}	301	14.95±0.58^{c}
G1G1(TTGG)	1120	816.47±213.93^{a}	1196	19.60±3.63^{a}	1196	6.35±0.70^{a}	1177	15.05±0.61^{b}
G1G2(CTGG)	550	780.71±171.35^{b}	582	19.27±3.65^{a}	582	6.30±0.71^{ab}	573	15.10±0.61^{ab}
G1G3(TTCG)	298	794.48±189.86^{ab}	307	17.97±4.10^{c}	307	6.25±0.76^{b}	303	15.02±0.58^{b}
G2G3(CTCG)	69	768.86±180.70^{ab}	70	17.27±3.93^{c}	70	6.06±0.56^{c}	67	15.21±0.64^{a}

G2G2(CCGG)	68	760.00±173.13[b]	69	18.95±4.22[ab]	69	6.37±0.68[ab]	68	15.22±0.69[a]
G3G3(TTCC)	20	756.51±101.13[ab]	21	17.22±3.44[bc]	21	6.17±0.56[abc]	21	15.03±0.61[ab]
H1H5(GACCGGGGGG)	125	847.79±237.45[a]	131	18.54±4.04[bc]	131	6.34±0.80[a]	130	15.07±0.59[bcde]
H1H4(GACCGGGGGG)	204	788.25±193.94[bcd]	217	18.71±3.78[b]	217	6.30±0.68[a]	213	15.17±0.63[abc]
H1H1(AACCGGGGGG)	670	812.48±211.65[ab]	713	19.49±3.71[a]	713	6.33±0.74[a]	704	15.00±0.60[e]
H1H3(GACCGGGGGG)	487	803.19±199.5[bc]	513	19.18±3.79[ab]	513	6.33±0.68[a]	503	15.08±0.62[cd]
H3H4(GAGCGGAGGG)	72	763.11±176.71[cd]	80	18.91±4.31[ab]	80	6.20±0.80[a]	78	15.23±0.57[ab]
H1H2(GACCAGGGGG)	213	777.46±149.57[cd]	223	19.62±3.57[a]	223	6.23±0.67[a]	220	15.04±0.60[de]
H3H3(GGCCGGGGGG)	89	782.53±161.69[bcd]	95	19.43±3.29[ab]	95	6.36±0.64[a]	93	15.05±0.53[cde]
H2H4(GACCGGGGGG)	36	745.84±186.79[cd]	35	19.02±4.17[abc]	35	6.38±0.54[a]	34	15.32±0.74[a]
H2H3(GACCGGGGGG)	73	744.92±148.61[d]	75	18.84±3.90[abc]	75	6.27±0.55[a]	74	15.00±0.56[de]
H3H5(GACCGGGGAG)	42	810.94±220.45[abcd]	44	17.46±4.18[c]	44	6.19±0.67[a]	43	14.99±0.74[cde]
I1I3(CCCCCCGT)	590	813.94±201.70[a]	619	18.42±3.93[b]	619	6.29±0.7[ab]	603	15.10±0.60[a]
I1I2(CCCCTCTT)	367	796.57±205.14[a]	389	20.00±3.39[a]	389	6.37±0.67[a]	383	15.06±0.60[a]
I2I2(CCCCTTTT)	154	806.40±225.24[a]	165	20.40±2.87[a]	165	6.37±0.72[ab]	164	15.03±0.56[a]
I1I1(112594424)	263	758.72±150.12[b]	276	18.05±3.75[b]	276	6.24±0.62[b]	273	15.08±0.63[a]
I3I3(CCCCCCGG)	66	846.45±190.24[a]	68	16.72±3.95[c]	68	6.02±0.80[c]	67	14.98±0.68[a]
J1J1(CCCCTT)	928	792.12±195.73[c]	981	19.25±3.65[ab]	981	6.27±0.68[c]	960	15.09±0.60[a]
J1J2(CCGCCT)	520	817.15±201.59[b]	549	19.10±3.97[b]	549	6.30±0.72[bc]	543	15.06±0.62[ab]
J1J3(TCGCCT)	438	788.00±187.32[c]	463	19.16±3.79[b]	463	6.36±0.72[b]	458	15.07±0.61[ab]
J2J2(CCGGCC)	88	868.84±246.67[a]	88	18.57±4.12[b]	88	6.26±0.75[bc]	87	15.02±0.65[ab]

J2J3(TCGGCC)	108	792.43±184.99^{bc}	116	19.04±3.71^{b}	116	6.51±0.75^{a}	114	14.97±0.62^{b}
J3J3(TTGGCC)	42	776.28±195.16^{bc}	47	20.33±3.53^{a}	47	6.47±0.84^{abc}	46	14.99±0.62^{ab}
K1K1(TTAA)	1175	810.14±202.61^{a}	1239	19.44±3.59^{a}	1239	6.33±0.71^{a}	1220	15.05±0.60^{b}
K1K2(GTGA)	805	794.34±196.33^{a}	856	19.01±3.93^{b}	856	6.31±0.72^{a}	841	15.07±0.63^{b}
K2K2(GGGG)	142	751.79±158.11^{b}	147	17.91±4.20^{c}	147	6.17±0.65^{b}	145	15.22±0.58^{a}
AA	449	821.61±199.86^{a}	480	18.88±4.04^{b}	480	6.37±0.75^{a}	473	15.20±0.65^{a}
AG	1111	799.39±197.56^{b}	1174	19.11±3.81^{b}	1174	6.32±0.73^{ab}	1152	15.07±0.61^{b}
GG	565	784.83±195.85^{b}	591	19.55±3.47^{a}	591	6.26±0.62^{b}	584	14.96±0.55^{c}

Abbreviations: CY= cashmere yield; FL= fiber length; CL= cashmere length; and CD= cashmere diameter. Values with the same within the same column mean no significant difference ($P > 0.05$), while with different letter superscripts mean significant difference ($P < 0.05$).
